# Investigating the impact of an efficacy-based nursing program integrated with nutritional intervention strategies on the outcomes of multiple myeloma patients with renal failure

**DOI:** 10.3389/fnut.2025.1623692

**Published:** 2025-08-12

**Authors:** Tingting Liu, Jing Lv, Luxiang Xu, Xiaomei Zhang, Qing Xiao, Jun Li, Zhaoli Zhang, Yao Liu

**Affiliations:** ^1^Chongqing Key Laboratory for the Mechanism and Intervention of Cancer Metastasis, Department of Hematology-Oncology, Chongqing University Cancer Hospital, Chongqing, China; ^2^Chongqing Key Laboratory for the Mechanism and Intervention of Cancer Metastasis, Nursing Department, Chongqing University Cancer Hospital, Chongqing, China

**Keywords:** multiple myeloma, renal failure, efficacy theory, nutritional intervention, nursing program

## Abstract

**Objective:**

To explore the effect of nursing scheme based on efficacy theory combined with nutritional intervention on patients with multiple myeloma (MM) complicated with renal failure.

**Methods:**

Ninety-two patients with MM complicated with renal failure in our hospital from April 2022 to April 2024 were randomly divided into control group (46 cases, using conventional nursing) and observation group (46 cases, using nursing scheme based on efficacy theory combined with nutritional intervention). The CDSES score of self-efficacy, CD-RISC score of resilience, CFS score of cancer-related fatigue, nutrition related indicators (ALB, Hb, PA) and FLIC score of quality of life were compared between the two groups.

**Results:**

After the intervention, both groups showed increased CDSES and CD-RISC scores, with the observation group significantly higher than controls. And the levels of ALB, Hb and PA in the observation group were significantly higher than those in the control group. Cancer fatigue scale (CFS) scores at T1, T2, and T3 were reduced from baseline (T0) in both groups; and the observation group was significantly lower than the control group. After the intervention, the scores of the four dimensions of good body and ability, good psychology, difficulties caused by cancer and good society of the two groups were significantly improved, and the scores of the observation group were higher than those of the control group; The scores of nausea dimension in the two groups were significantly lower than those before the intervention, and the control group decreased more than the observation group (*p* < 0.05).

**Conclusion:**

The efficacy theory-based nursing plan combined with nutritional intervention benefits MM patients with renal failure. It improves self-efficacy and psychological resilience, reduces cancer-related fatigue, and enhances nutritional status and overall quality of life.

## 1 Introduction

Multiple myeloma (MM), a malignant tumor originating from plasma cells, is often accompanied by osteolytic lesions, hypercalcemia, anemia, and other symptoms. It accounts for approximately 1.5% of all cancer cases and constitutes approximately 10% of hematologic malignancies (1). The immunoglobulins and their components (such as light chains) secreted by myeloma tumor cells can form casts that obstruct renal tubules. Additionally, as the cancer cells progress, they may infiltrate the kidneys, resulting in renal tissue damage and ultimately leading to renal failure (2, 3). The current clinical strategies for the treatment of multiple myeloma (MM) complicated with renal failure primarily rely on medications such as bortezomib and lenalidomide. These agents effectively delay disease progression by inhibiting the proliferation of myeloma cells. However, long-term pharmacological treatment is accompanied by complex side effects. Coupled with patients’ limited disease awareness and insufficient self-management capabilities, these factors often lead to diminished confidence during treatment, fostering negative emotions such as fear and anxiety, which ultimately compromise therapeutic efficacy and quality of life (4). Consequently, there is an urgent need to explore effective nursing interventions and nutritional support programs to complement drug therapy, enhance patients’ self-efficacy and psychological resilience.

Efficacy theory, a psychological framework emphasizing the interaction between individual practice and cognition, posits that self-efficacy—an individual’s belief in their ability to complete specific tasks or achieve goals—serves as a crucial driver for proactive behaviors when facing challenges (5). In oncology nursing, the application of efficacy theory aims to help patients build confidence in disease management and improve self-management capabilities through education, guidance, and support, thereby effectively addressing physical and psychological challenges during treatment. Nutritional intervention, as an essential component of comprehensive cancer care, plays an irreplaceable role in improving nutritional status, enhancing immunity, and alleviating treatment-related side effects. MM patients with renal failure frequently experience severe nutritional issues due to disease-related appetite loss and metabolic abnormalities exacerbated by treatment, further accelerating functional decline (6). Targeted nutritional interventions therefore seek to address patients’ dietary needs through scientifically designed meal plans to support physical recovery.

The integration of efficacy theory with nutritional interventions in MM-renal failure patient care demonstrates dual necessity: On one hand, efficacy theory-guided approaches enhance patients’ self-efficacy in disease management, strengthening their confidence to actively engage in treatment. On the other hand, nutritional interventions improve physiological reserves critical for recovery. These complementary strategies synergistically address patients’ physical and mental health needs, potentially providing comprehensive and personalized care to optimize quality of life and accelerate rehabilitation. For patients with renal failure, research suggests that a multidisciplinary approach to nutrition management should be provided by providing nutritional assessment and support, dietary counseling, management of comorbid conditions, and by maintaining an adequate dialysis dose and preserving residual renal function (7). Meanwhile, nurses should use multiple strategies based on self-efficacy theory to improve patients’ self-efficacy levels and self-management capacities to improve the health status of patients with renal failure (8). However, it has not been reported whether the efficacy theory-based nursing protocol combined with nutritional interventions can bring further clinical benefits to MM patients with renal failure. This study will therefore investigate the impact of an efficacy theory-based nursing protocol combined with nutritional interventions on MM patients with renal failure.

## 2 Materials and methods

### 2.1 General information

Between April 2022 and April 2024, 92 patients with MM renal failure were admitted to our hospital. They were randomly divided into a control group (46 cases, receiving routine care) and an observation group (46 cases, receiving therapeutic theory care combined with nutritional intervention) using the computer-generated random number table method. Baseline characteristics showed no significant differences between the two groups (*p* > 0.05, Table 1).

**Table 1:** Baseline characteristics comparison between groups.

Group	*n*	Gender [*n* (%)]		Age (years)	BMI (kg/m^2^)	Education (years)	Pathological Stage [*n* (%)]		
		Male	Female				I	II	III
Observation	46	29 (63.04)	17 (36.96)	54.2 ± 6.2	22.58 ± 1.79	9 ± 4	20 (43.48)	18 (39.13)	8 (17.39)
Control	46	30 (65.22)	16 (41.30)	56.0 ± 6.2	22.06 ± 1.97	10 ± 4	21 (45.65)	18 (39.13)	7 (15.22)
*χ*^2^/*t*	–	0.047		−1.397	1.329	−0.666	0.091		
*P*	–	0.828		0.166	0.187	0.507	0.955		

BMI, body mass index.

#### 2.1.1 Inclusion criteria

1. All patients met the diagnostic criteria for MM complicated by renal failure, as defined by the Diagnosis and Treatment Guidelines for Multiple Myeloma (2018 Edition) (9) and Clinical Guidelines for Chronic Renal Failure (3);
2. Ability to perform independent activities and cooperate with the study, including follow-up;
3. Diagnosis and treatment conducted at our hospital, with no prior chemotherapy or antitumor therapy before study initiation;
4. Availability of complete baseline medical records.

#### 2.1.2 Exclusion criteria

1. Concurrent infections, hematological disorders, or other malignancies;
2. Cognitive impairment or psychiatric disorders;
3. Cardiac or cerebral insufficiency;
4. Comorbidities involving other plasma cell proliferative diseases, such as severe infections, anemia, hypercalcemia, etc.

This study was approved by the hospital ethics committee.

### 2.2 Methods

#### 2.2.1 Control group

The control group received routine care, implemented as follows:

1.  Dietary management: Provided a high-calorie, high-vitamin, low-salt, low-fat, high-quality low-protein, and easily digestible diet to reduce renal burden while meeting energy requirements.
2.  Pain and fluid management: Conducted pain assessments twice daily (morning and afternoon) and adjusted pain management protocols based on results. Fluid intake and output were recorded every 4 h to maintain fluid balance.
3.  Infection prevention:

- Performed ultraviolet (UV) irradiation disinfection of indoor air twice daily (30 min per session).
- Cleaned surfaces, furniture, and floors daily using chlorine-containing disinfectant.
- Restricted visitor access and delivered weekly infection control education.

4.  Condition monitoring:

- Recorded vital signs hourly, fluid balance, and body weight daily.
- Performed blood urea nitrogen (BUN) and serum creatinine tests twice weekly to evaluate disease progression.

5.  Medication management:

- Provided medication guidance twice daily (morning and afternoon) to ensure adherence to prescribed regimens, with detailed documentation.
- Conducted monthly medication education sessions to enhance compliance.

6.  Psychological support:

- Performed psychological assessments twice weekly to evaluate mental status and deliver tailored counseling.
- Encouraged family involvement in psychological care to foster a positive mindset.

7.  Exercise guidance:

- Supervised light exercise (e.g., non-strenuous activities) under physician or nurse guidance, emphasizing gradual progression and avoidance of heavy lifting.

#### 2.2.2 Observation group

The observation group received efficacy theory combined with nutritional intervention on the basis of the control group.

##### 2.2.2.1 Efficacy theory nursing model

###### 2.2.2.1.1 Intervention team formation

An intervention team was established, consisting of the head nurse, department director, nutritionist, attending physician, specialized nutrition nurse, psychologist, and assigned nurses. The head nurse served as the team leader, convening weekly meetings to discuss patient care progress and existing issues. The team leader organized monthly training sessions on nutritional care for MM with renal failure and efficacy theory. Assessments of patients’ physical/mental status and self-efficacy were conducted to evaluate their confidence and capabilities in disease management, nutritional intake, and medical compliance. An intervention plan was formulated, with implementation supervised by assigned nurses.

###### 2.2.2.1.2 Goal setting

Efficacy theory emphasizes setting clear, achievable goals for individuals and providing appropriate incentives and support during the process. In practice, the team collaborated with patients to set specific, realistic care goals—such as daily nutritional intake and exercise plans—based on self-efficacy assessments. Goals were designed to align with patients’ values and expectations while remaining feasible. Weekly evaluations ensured progress toward goal attainment.

###### 2.2.2.1.3 Intervention plan implementation

Assigned nurses used animated videos, PPT presentations, and WeChat infographics to explain disease characteristics, treatment plans, and self-management strategies, enhancing patients’ knowledge, skills, and confidence. Patients were encouraged to self-monitor and report outcomes to visualize their progress. Psychologists conducted biweekly counseling sessions, employing positive communication to rebuild patients’ self-confidence and guide them toward autonomous self-management. Continuous positive feedback and support were provided. A family support and rehabilitation plan was co-developed with patients to strengthen their social support system. Monthly rehabilitation (exchange meetings) facilitated peer interaction, experience sharing, and emotional bonding to boost morale.

###### 2.2.2.1.4 Feedback and adjustment

Complication rates and behavioral changes were assessed. The care plan was dynamically adjusted based on patient feedback and practical outcomes to ensure goal achievement.

##### 2.2.2.2 Nutritional intervention

###### 2.2.2.2.1 Nutritional assessment

Weekly evaluations of nutritional status—including weight, dietary habits, and biochemical indicators—were conducted to identify needs and issues.

###### 2.2.2.2.2 Dietary planning

Personalized meal plans were tailored to patients’ nutritional requirements and clinical conditions. Recommendations included high-calorie, high-vitamin, low-salt, low-fat, and high-quality low-protein foods, while restricting phosphorus-, potassium-, and purine-rich items.

###### 2.2.2.2.3 Nutritional support

Individualized support strategies were implemented. Patients with poor appetite or insufficient intake received daily oral nutritional supplements or intravenous support. Weekly monitoring guided adjustments to dietary plans. Patients were encouraged to consume iodine-, iron-, calcium-, and vitamin-rich foods (e.g., kelp, laver, almonds, pork liver, walnuts) to enhance bodily functions, blood production, and immunity. Strict monitoring of salt, protein, and fluid intake was enforced, alongside calorie/nutrient supplementation (daily energy intake of 35 kcal/kg for patients on peritoneal dialysis <60 years and 30–35 kcal/kg for patients >60 years and daily protein intake of 1.2–1.3 g/kg) (10). Irritating foods were discouraged. Nutritional support continues until the patient reaches the terminal stage where they are unable to eat.

###### 2.2.2.2.4 Education and communication

Weekly nutritional education sessions provided patients and families with detailed guidance on food selection, cooking methods, and nutrient importance. Open dialog was encouraged to address questions and suggestions, fostering two-way communication.

### 2.3 Observation indicators

#### 2.3.1 Self-efficacy comparison

Self-efficacy was assessed using the Chronic Disease Self-efficacy Scale (CDSES) (11). Patients were evaluated before and after the intervention. Developed by psychologist Albert Bandura at Stanford University in the 1970s, the CDSES measures an individual’s confidence in their ability to execute specific behaviors in particular contexts and their belief in achieving goals within a defined domain. The scale comprises 33 items across four dimensions: general self-management, task completion, problem-solving, and outcome achievement. Each item is scored from 0 to 10, with higher scores indicating stronger self-efficacy.

#### 2.3.2 Psychological resilience comparison

The Connor-Davidson Resilience Scale (CD-RISC) (12) was used to assess psychological resilience before and after the intervention. Developed by Katherine M. Connor and Jonathan R.T. Davidson, this scale evaluates an individual’s capacity to adapt to adversity and stress. It includes 25 items across three dimensions (strength, tenacity, and optimism), each rated on a 0–4 scale. Higher total scores reflect greater psychological resilience and better stress-coping abilities.

#### 2.3.3 Cancer-related fatigue comparison

Cancer-related fatigue was measured using the Chinese version of the Cancer-Related Fatigue Scale (CFS) (13), adapted from the original by Okuyama et al. (14). The scale contains 15 items across three dimensions (physical, emotional, and cognitive), with a total score of 60 points. Each item employs a 5-point Likert scale (1 = “not at all,” 2 = “a little,” 3 = “somewhat,” 4 = “quite a bit,” 5 = “extremely”), where higher scores indicate more severe fatigue. Scores were recorded and compared at baseline, 1 week, 1 month, and 3 months post-intervention.

#### 2.3.4 Nutritional indicator comparison

Fasting blood samples (5 mL of antecubital venous blood) were collected in the morning and centrifuged to obtain serum. An automated biochemical analyzer was used to measure nutritional markers, including serum albumin (ALB), hemoglobin (Hb), and prealbumin (PA).

#### 2.3.5 Quality of life comparison

The Functional Living Index-Cancer (FLIC) (15), developed by Schipper in 1984, was used to assess quality of life. This self-reported scale includes 22 items across five domains: psychological, physical, social functioning, cancer-related hardship, and nausea. Each item is scored from 1 to 7, with higher total scores indicating better quality of life. Patients rated each item based on their actual experiences.

### 2.4 Statistical analysis

All data in this study were analyzed using SPSS 22.0 software. Categorical data were expressed as n (%), and intergroup comparisons were performed using the chi-square test. Normally distributed continuous data were presented as mean ± standard deviation, with intergroup comparisons conducted via the independent t-test and intragroup pre- and post-intervention comparisons via the paired t-test. A *p*-value < 0.05 was considered statistically significant.

## 3 Results

### 3.1 Comparison of CDSES scores between groups before and after intervention

**Figure 1:**
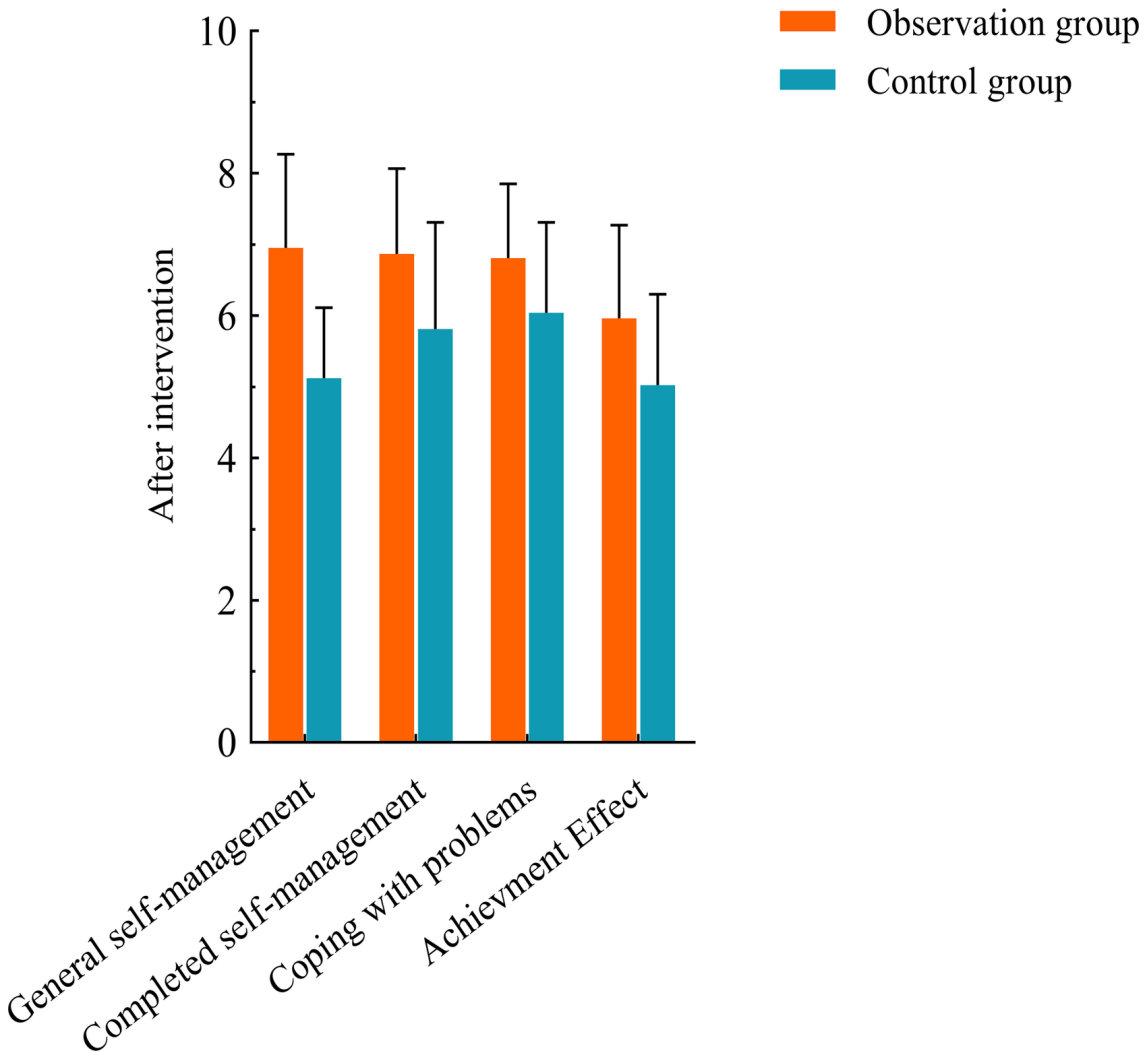
Comparison of CDSES scores between the two groups before and after intervention. Compared with control group, ^*^p < 0.05.

**Figure 2:**
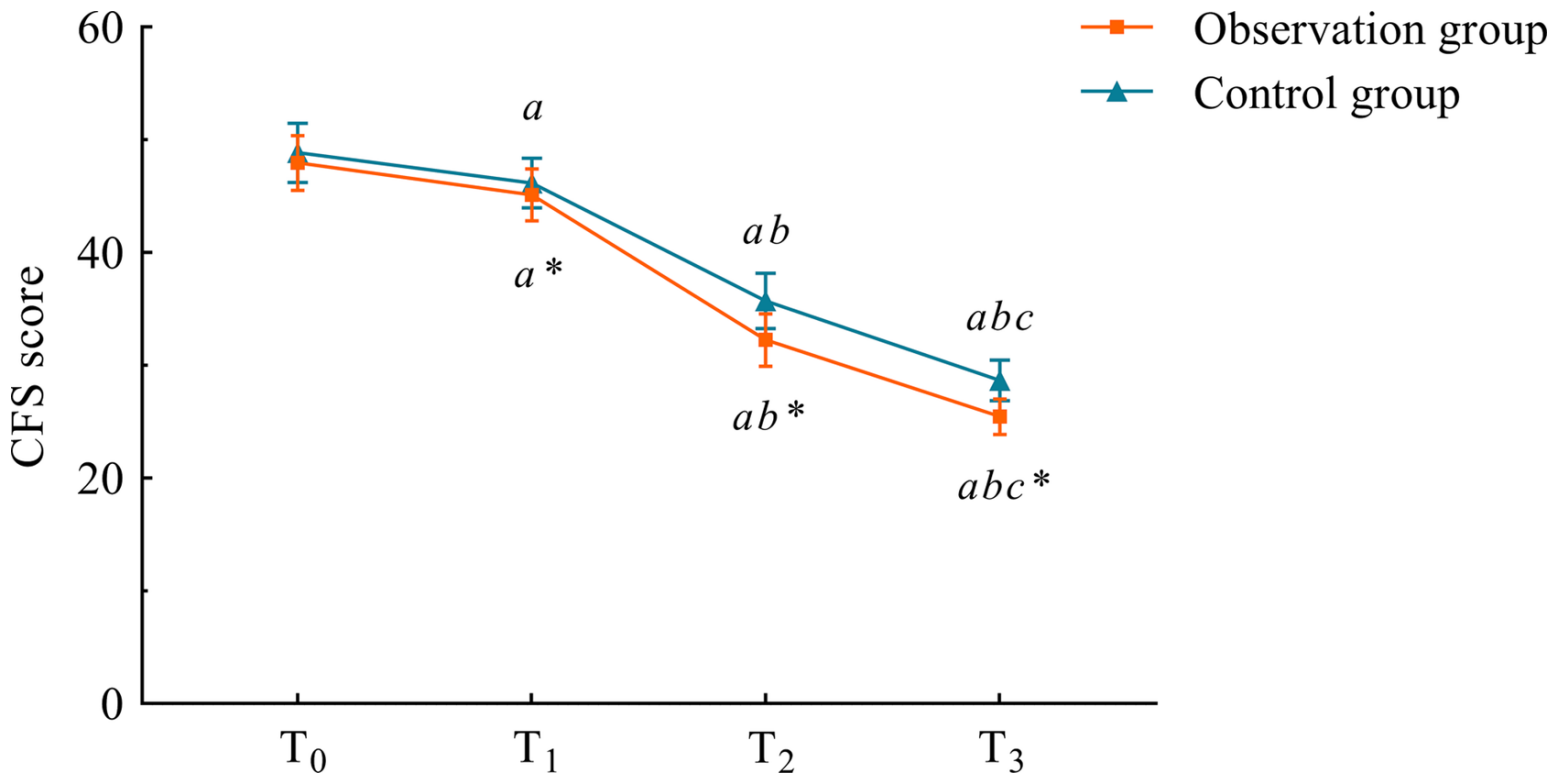
Trends of CFS scores at different time points in the two groups. Compared with the control group, ^*^p < 0.05. Compared with T0, ^a^*p* < 0.05; compared with T1, ^b^*p* < 0.05; compared with T2, ^c^*p* < 0.05. T0, T1, T2, and T3 mean time points.

No significant differences were observed between the two groups before the intervention (*p* > 0.05). After the intervention, both groups showed increased scores in general self-management, task completion, problem-solving, and outcome achievement compared to baseline. However, the observation group demonstrated significantly higher scores than the control group (*p < 0.05) (see Table 2; Figures 1, 2 for details).

**Table 2:** Comparison of CDSES scores between the two groups before and after intervention.

Index	Group	*n*	Before intervention	After intervention	Paired *t*-value	Paired *p*-value
General self-management	Observation group	46	3.74 ± 1.05	6.95 ± 1.32	−12.898	<0.001
	Control group	46	3.45 ± 1.15	5.12 ± 0.99	−7.472	<0.001
	Independent *t*-value	–	1.242	7.512	–	–
	Independent *p*-value	–	0.218	<0.001	–	–
Completed self-management	Observation group	46	3.23 ± 1.44	6.87 ± 1.20	−13.162	<0.001
	Control group	46	3.58 ± 1.29	5.81 ± 1.50	−7.652	<0.001
	Independent *t*-value	–	−1.219	3.722	–	–
	Independent *p*-value	–	0.226	<0.001	–	–
Coping with problems	Observation group	46	3.79 ± 1.17	6.81 ± 1.04	−12.202	<0.001
	Control group	46	3.59 ± 1.19	6.04 ± 1.27	−9.549	<0.001
	Independent *t*-value	–	0.800	3.185	–	–
	Independent *p*-value	–	0.426	0.002	–	–
Achievement effect	Observation group	46	2.41 ± 0.51	5.96 ± 1.31	−15.943	<0.001
	Control group	46	2.32 ± 0.47	5.02 ± 1.28	−13.433	<0.001
	Independent *t*-value	–	0.859	3.493	–	–
	Independent *p*-value	–	0.393	0.001	–	–

### 3.2 Comparison of CD-RISC scores between the two groups before and after intervention

Before the intervention, there were no significant differences between the two groups in terms of strength, optimism, or resilience (*p* > 0.05). After the intervention, both groups showed an increase in CD-RISC scores compared to pre-intervention levels, with the observation group demonstrating significantly higher scores than the control group (*p* < 0.05) (see Table 3).

**Table 3:** Comparison of CD-RISC scores between the two groups before and after intervention.

Index	Group	*n*	Before intervention	After intervention	Paired *t*-value	Paired *p*-value
Strength	Observation group	46	18.42 ± 2.37	26.36 ± 2.64	−15.184	<0.001
	Control group	46	19.14 ± 2.49	22.24 ± 2.17	−6.376	<0.001
	Independent *t*-value	–	−1.403	8.174	–	-
	Independent *p*-value	–	0.164	<0.001	–	–
Optimism	Observation group	46	8.65 ± 1.29	13.75 ± 1.66	−16.430	<0.001
	Control group	46	8.28 ± 1.29	11.58 ± 1.67	−10.592	<0.001
	Independent *t*-value	–	1.360	6.244	–	–
	Independent *p*-value	–	0.177	<0.001	–	–
Resilience	Observation group	46	27.28 ± 3.65	41.95 ± 4.28	−17.705	<0.001
	Control group	46	28.22 ± 3.64	36.79 ± 4.79	−9.653	<0.001
	Independent *t*-value	–	−1.244	5.452	–	–
	Independent *p*-value	–	0.217	<0.001	–	–

### 3.3 Comparison of CFS scores between the two groups at different time points

There was no difference in CFS scores between the two groups at the T0 time point (*p* > 0.05). However, at all post-intervention assessments (1 week, 1 month, and 3 months after the intervention), scores in both groups were lower than the pre-intervention T0 scores, with the observation group showing significantly lower scores compared to the control group (*p* < 0.05) (Table 4; Figure 2).

**Table 4:** Comparison of CFS scores between the two groups at different time points.

Group	*n*	T0	T1	T2	T3
		Before intervention	1 Week after intervention	1 Month after intervention	3 Months after intervention
Observation group	46	47.97 ± 2.42	45.13 ± 2.29^a*^	32.26 ± 2.32^ab*^	25.46 ± 1.57^abc*^
Control group	46	48.85 ± 2.63	46.18 ± 2.21^a^	35.74 ± 2.45^ab^	28.68 ± 1.79^abc^
*t*	–	−1.675	−2.224	−7.004	−9.195
*p*	–	0.097	0.029	<0.001	<0.001
*F*	–	*F*_time_ = 1801.339; *F*_group_ = 91.932; *F*_time*group_ = 8.629			
*p*	–	*p*_time_<0.001; *p*_group_<0.001; *p*_time*group_<0.001			

Compared with the control group, ^a^*p* < 0.05. Compared with T0, ^b^*p* < 0.05. Compared with T1, ^c^*p* < 0.05. Compared with T2, ^d^*p* < 0.05. T0, T1, T2, and T3 mean time points.

### 3.4 Comparison of nutritional indicators between the two groups before and after intervention

Before the intervention, there were no statistically significant differences in nutritional indicators (ALB, Hb, PA) between the two groups (*p* > 0.05). After the intervention, levels of ALB, Hb, and PA in both groups increased compared to pre-intervention levels (*p* < 0.05), with the observation group exhibiting significantly higher levels of ALB, Hb, and PA than the control group (*p* < 0.05) (see Table 5).

**Table 5:** Comparison of nutritional indicators between the two groups before and after intervention.

Index	Group	*n*	Before intervention	After intervention	Paired *t*-value	Paired *p*-value
ALB (g/L)	Observation group	46	33.37 ± 4.52	49.71 ± 5.22	16.352	<0.001
	Control group	46	33.41 ± 5.04	44.61 ± 6.03	10.375	<0.001
	Independent *t*-value	–	0.040	4.337	–	–
	Independent *p*-value	–	0.968	<0.001	–	–
Hb (g/L)	Observation group	46	81.83 ± 7.52	114.01 ± 10.33	20.095	<0.001
	Control group	46	82.28 ± 8.93	91.62 ± 11.23	4.282	<0.001
	Independent *t*-value	–	0.267	9.948	–	–
	Independent *p*-value	–	0.790	<0.001	–	–
PA (mg/L)	Observation group	46	249.56 ± 21.09	338.83 ± 34.39	17.662	<0.001
	Control group	46	251.32 ± 23.04	307.14 ± 35.24	8.464	<0.001
	Independent *t*-value	–	0.382	4.367	–	–
	Independent *p*-value	–	0.703	<0.001	–	–

### 3.5 Comparison of FLIC scores between the two groups before and after intervention

After the intervention, both groups showed significant improvements in scores across the four dimensions of physical well-being and capacity, psychological well-being, hardship caused by cancer, and social well-being compared to pre-intervention levels, with the observation group demonstrating higher scores than the control group. In contrast, scores in the nausea dimension were significantly lower than pre-intervention levels in both groups, with the control group exhibiting a greater reduction compared to the observation group (*p* < 0.05) (see Table 6).

**Table 6:** Comparison of FLIC scores between the two groups before and after intervention.

Index	Group	*n*	Before intervention	After intervention	Paired *t*-value	Paired *p*-value
Somatic well-being and ability	Observation group	46	38.34 ± 7.22	46.38 ± 5.20	−6.127	<0.001
	Control group	46	37.30 ± 7.54	41.96 ± 6.90	−3.096	0.003
	Independent *t*-value	–	0.676	3.466	–	–
	Independent *p*-value	–	0.500	<0.001	–	–
Psychological well-being	Observation group	46	26.83 ± 3.52	34.01 ± 3.33	−10.047	<0.001
	Control group	46	26.28 ± 3.28	31.62 ± 3.23	−7.874	<0.001
	Independent *t*-value	–	0.775	3.486	–	–
	Independent *p*-value	–	0.440	<0.001	–	–
Difficulties caused by illness	Observation group	46	13.61 ± 1.71	18.07 ± 2.19	−10.889	<0.001
	Control group	46	13.16 ± 1.17	15.44 ± 2.01	−6.640	<0.001
	Independent *t*-value	–	1.477	6.001	–	–
	Independent *p*-value	–	0.143	<0.001	–	–
Social well-being	Observation group	46	8.96 ± 0.89	12.46 ± 1.03	−17.430	<0.001
	Control group	46	8.66 ± 0.96	10.76 ± 1.13	−9.630	<0.001
	Independent *t*-value	–	1.595	7.565	–	–
	Independent *p*-value	–	0.114	<0.001	–	–
Nausea	Observation group	46	12.55 ± 1.09	9.35 ± 0.83	15.844	<0.001
	Control group	46	12.32 ± 1.02	8.44 ± 0.72	20.941	<0.001
	Independent *t*-value	–	1.069	5.592	–	–
	Independent *p*-value	–	0.288	<0.001	–	–

## 4 Discussion

Multiple myeloma (MM), as a plasma cell malignancy, poses a severe threat to patients’ physical function due to its complex pathophysiological mechanisms. The challenge of treatment is significantly amplified when MM is complicated by renal failure. The clonal immunoglobulins produced by abnormal proliferation of MM cells not only impair normal bone marrow function but also damage renal function through tubular obstruction and toxic effects. Concurrently, hypercalcemia and elevated uric acid levels further exacerbate renal deterioration (16, 17). These pathological changes not only restrict physiological functions but also profoundly impact patients’ psychological state and social functioning, leading to a severe decline in the quality of life for MM patients with renal failure (18).

Currently, although medications, chemotherapy, and hemodialysis play critical roles in managing MM with renal failure, challenges such as long-term chemotherapy resistance and poor adherence to hemodialysis cannot be overlooked. Prolonged use of chemotherapeutic agents may induce drug resistance in tumor cells, limiting sustained therapeutic efficacy. While hemodialysis temporarily alleviates renal burden, its effectiveness heavily depends on patients’ regular participation and compliance, which may result in suboptimal outcomes and complications (19, 20). These factors impose high demands on patients’ self-management capabilities. Therefore, exploring a comprehensive nursing approach that enhances self-efficacy, improves psychological well-being, and optimizes nutritional status is imperative.

The core of the efficacy theory-based nursing model lies in establishing personalized, achievable care goals tailored to individual patient conditions and facilitating goal attainment through continuous motivation and support (21). In this study, the formation of a multidisciplinary intervention team enabled comprehensive physical and psychological assessments of patients, leading to individualized care plans. This patient-centered approach not only enhanced the precision and effectiveness of nursing interventions but also fostered effective communication between healthcare providers and patients, improving trust and engagement.

Goal setting is pivotal in the efficacy theory-based nursing model. Collaboratively establishing specific, achievable targets—such as daily nutritional intake and exercise plans—helped patients build confidence and accountability in disease management. These goals reflect patients’ values and expectations, motivating active participation in self-care. During implementation, diverse educational tools, including animated videos and PPT presentations, were utilized to explain disease characteristics, treatment protocols, and key self-management strategies, significantly improving patients’ knowledge and self-management skills (22).

Nutritional intervention is equally vital for MM patients with renal failure. Disease progression and treatments often predispose patients to malnutrition, which compromises therapeutic efficacy and exacerbates fatigue and psychological distress. Personalized dietary plans were designed based on patients’ nutritional needs and clinical profiles, emphasizing high-calorie, high-vitamin, low-salt, low-fat, and high-quality low-protein foods while restricting phosphorus-, potassium-, and purine-rich items. These adjustments alleviated renal burden, met energy requirements, and optimized nutritional balance (23). Oral or intravenous nutritional supplements were provided for patients with poor appetite or inadequate intake. Nutritional education and regular communication with patients and families reinforced understanding and practical application of dietary management.

This study demonstrated that the efficacy theory-based nursing model combined with nutritional intervention significantly improved outcomes in MM patients with renal failure. Compared to the control group, the observation group exhibited notably higher self-efficacy (CDSES) and psychological resilience (CD-RISC) scores, indicating enhanced confidence in disease management. Cancer-related fatigue (CFS), a multidimensional condition involving physical decline, psychological distress, and social limitations, was significantly reduced in the observation group, suggesting effective alleviation of fatigue (24, 25). Nutritional markers, including ALB, Hb, and PA levels, were superior in the observation group, reflecting improved nutritional status. These enhancements likely bolstered immune function, treatment tolerance, and recovery. Additionally, the observation group scored higher across most quality-of-life domains (physical function, psychological well-being, and social interaction) except for nausea, which may relate to chemotherapy-induced gastrointestinal discomfort. These improvements underscore the holistic benefits of the intervention on patients’ physiological, psychological, and social well-being (26).

Despite these promising results, limitations exist. The relatively small sample size may restrict generalizability, necessitating larger-scale studies to validate efficacy and safety. As a prospective study, long-term follow-up data are lacking to assess sustained impacts. Future research should extend follow-up periods to evaluate long-term quality-of-life outcomes. Furthermore, while this study focused on the effects of efficacy theory-based nursing and nutritional interventions on self-efficacy, psychological resilience, fatigue, and nutritional status, deeper exploration of underlying mechanisms is warranted. Investigating interactions among these factors and their pathways to improving treatment outcomes and quality of life would provide valuable insights.

## 5 Conclusion

The efficacy theory-based nursing model combined with nutritional intervention demonstrates significant potential in enhancing self-efficacy, psychological resilience, nutritional status, and overall quality of life for MM patients with renal failure. These findings highlight the importance of integrating personalized care strategies and multidisciplinary collaboration in managing complex hematological malignancies.

## Data Availability

The original contributions presented in the study are included in the article/supplementary material, further inquiries can be directed to the corresponding author/s.
